# Expression and potential role of major inflammatory cytokines in experimental keratomycosis

**Published:** 2009-07-04

**Authors:** Wenxian Zhong, Hongmei Yin, Lixin Xie

**Affiliations:** State Key Laboratory Cultivation Base, Shandong Provincial Key Laboratory of Ophthalmology, Shandong Eye Institute, Qingdao, China

## Abstract

**Purpose:**

The aim of this study was to investigate the expression and regulation of the four major inflammatory cytokines in fungal keratitis (FK) with the goal of further understanding its pathogenesis in order to develop more effective therapeutic approaches.

**Methods:**

*Aspergillus fumigatus* and *Candida albicans* were the corneal pathogens selected for this study to establish murine FK using epikeratophakia with the aid of corneal epithelium erasion. One, three, five, and seven days post-infection, the corneal lesions and inflammatory responses were observed by slit-lamp and histopathology, and the expressions of the four inflammatory cytokines, macrophage inflammatory protein-2 (MIP-2), cytokine-induced neutrophil chemoattractant (KC), interleukin-1β (IL-1β), and interleukin-6 (IL-6), in the infected corneas were determined using reverse transcription polymerase chain reaction (RT–PCR) and enzyme-linked immunosorbent assay (ELISA). For the intervention experiment with neutralizing antibodies, the experimental mice were then injected subconjunctivally with 5 μl (2 ng/μl) MIP-2 or IL-1β polyclonal antibody 1 h before and 24 h after surgery. Reestablishment of the FK murine model was performed following injection. Effects of MIP-2 or IL-1β polyclonal antibody on the corneal diseases were observed by slit-lamp microscopy, histopathology, and ELISA.

**Results:**

Expression of MIP-2, KC, IL-1β, and IL-6 was upregulated significantly in the infected group one, three, five, and seven days after surgery. Following treatment with an MIP-2 polyclonal antibody, the corneal clinical scores and inflammatory responses decreased, the MIP-2 protein levels were downregulated significantly (p<0.01), and the KC protein levels decreased slightly (p>0.05). Upon administration of IL-1β polyclonal antibodies, the decrease in clinical scores, inflammatory responses, and protein levels of MIP-2 and KC was apparent at 1 and 3 days after infection (p<0.01).

**Conclusions:**

A persistent, high level expression of MIP-2 and IL-1β is an important and even major factor in the corneal pathogenesis of FK. Specific polyclonal neutralizing antibodies may be administered to inhibit the major chemokines and cytokines responsible for corneal damage thus effectively relieving the injury caused by FK.

## Introduction

The incidence of fungal keratitis (FK) is increasing in many agricultural countries over the past few decades, and it is even the primary cause for corneal blindness in some nations [1-4]. The most common pathogens responsible for FK in most countries are *Fusarium* spp. and *Aspergillus* spp., but *Fusarium solani* and *Aspergillus fumigatus* are also commonly seen [2,3,5,6]. However, the most common pathogen in the majority of Europe and America is *Candida albicans* with an exception being the southern region of the United States [7,8]. FK is characterized by a serious, suppurative inflammatory process. In addition to fungal virulence factors, which include invasiveness, toxigenicity, and proteolytic enzymes, the host inflammatory response is perhaps the most important cause of the destruction seen with these corneal lesions [9]. In histopathological studies of FK patients and experimental animals, it was determined that the majority of inflammatory cells present in corneal tissue with FK are polymorphonuclear neutrophils (PMNs) [10,11].

The studies have demonstrated that purulent inflammation is primarily mediated by specific inflammatory cytokines including chemokines and cytokines [12,13]. The PMNs are primarily recruited by chemokines including IL-8 (mouse homolog macrophage inflammatory protein-2 [MIP-2]) and growth-related oncogene-α (GRO-α, mouse homolog KC) [13]. The role of MIP-2 is more important than KC in promoting the migration of PMNs [14]. The functions of IL-1β include the regulation of acute phase response, chemotaxis, activation of inflammatory cell, and irritation of neovascularization [15]. Furthermore, the expression of MIP-2 and KC is predominantly regulated by IL-1β [16]. IL-6 is considered to play an important protective role in the infected keratitis [17]. Thus, we presumed that pathogenic fungi and/or host cells’ production of cytokines would be responsible for the suppurative inflammation characteristic of FK. However, few correlative studies of FK have been published [18,19]. To confirm the role of four major inflammatory cytokines (MIP-2, KC, IL-1β, IL-6) in FK, this study established a keratomycosis model using inbred BALB/c mice. Then, at different time points after inoculation, expression of the four inflammatory cytokines (MIP-2, KC, IL-1β, and IL-6) in the infected cornea was detected at the mRNA and protein level. Based on the results of the cytokine expression studies, polyclonal antibodies against the responsible cytokines were administered to neutralize the inflammatory effects, and changes in the corneal lesion, histopathology, and expression of major inflammatory cytokines in the infected cornea were observed.

## Methods

### Animal and pathogen

Six- to eight-week-old inbred BALB/c mice of either sex, weighing 18–20 g each, were used in this study. No disease was found in these animals by slit-lamp examination and indirect funduscopy. All animals were treated in accordance with the Association for Research in Vision and Ophthalmology Statement for the Use of Animals in Ophthalmic and Vision Research. Corneal infections were induced in the right eye.

The strains of *Aspergillus fumigatus* and *Candida albicans* were AS 3.0772 (China Committee of Culture Collection for Microorganisms, Beijing, China) and ATCC MYA-2876 (American Type Culture Collection, Rockville, MD), which were originally isolated from human skin and a patient with generalized candidiasis, respectively. The fungal isolate was cultured on Sabouraud glucose agar at 28 °C for five days, the colony was flushed from the dish with 0.1% Tween-20 solution, and the suspension was centrifuged at 500x g for 5 min. The sedimented spores were harvested in 1 ml of sterile saline solution and then diluted with sterile saline to yield 10^6^ CFU/ml.

### Murine model of fungal keratitis

The fungal keratitis model was prepared according to the previous report [11] with a minor modification. Briefly, the mice were anesthetized with a ketamine (50 mg/kg) and chlorpromazine hydrochloride (10 mg/kg) intraperitoneal injection, and 0.4% oxybuprocaine eye drops were used topically for corneal anesthesia. The central corneal epithelium was removed in a diameter of 2 mm before a full thickness rat corneal button was placed on the mouse cornea. Inoculum (5 μl; spores, 10^6^ culturable units) of *A. fumigatus* and *C. albicans* was injected into the space between the two corneas. The eyelid was then sutured with two interrupted 10–0 nylon suture to secure the rat corneal button, and another 5 μl of inoculum was injected into the conjunctival sac. Immediately after surgery, 0.3% ofloxacin eye ointment (Tarivid; Santen, Osaka, Japan) was administered to the palpebral margin. For the intervention experiment with neutralizing antibodies, mice were subconjunctivally injected with 5 μl (2 ng/μl) of MIP-2 polyclonal antibody (MIP-2 treated group), IL-1β polyclonal antibody (IL-1β treated group), or sterile saline (infected group) both 1 h before and 24 h after surgery. Reestablishment of the FK murine model was performed following injection. The eyelid suture and the corneal graft were removed 24 h after surgery. Experimental eyes were treated with 0.3% ofloxacin eye drops four times a day. One, three, five, and seven days post infection, the mice were photographed and scored clinically before being sacrificed. The eyes were enucleated and processed for histological examination, polymerase chain reaction (PCR) analysis, and enzyme-linked immunosorbent assay (ELISA).

### Clinical scoring

The inoculated eyes were monitored daily, and the severity of keratomycosis was scored with the aid of a dissecting microscope at one, three, five, and seven days. A grading of 0−4 was assigned to the area of opacity, density of opacity, and surface regularity. The scores from these three categories were tallied for each eye to yield a total score ranging from 0−12; 1−5 being for mild eye disease, 5−9 for moderate, and >9 for severe [20].

### Histology

One, three, five, and seven days after surgery, the enucleated eyeballs were immediately fixed in formalin, dehydrated with ethanol, and paraffin embedded. Continuous 4 μm sections were stained with hematoxylin-eosin (H&E) and periodic acid–Schiff (PAS). The presence of inflammatory cells and fungal hyphae was observed by light microscopy.

The presence or absence of inflammatory cells, the type of cells, the degree of inflammation, depth, and distribution of the cells were noted. The degree of inflammation was graded (in a semi-quantitative way) under a microscope in a masked fashion as mild, moderate, or severe based on the density of inflammatory cells in the corneal tissues [10]. Inflammation was considered mild if the background stroma and keratocyte nuclei were seen clearly; moderate if the keratocyte nuclei were obliterated, but the stroma was visible; and severe if there was complete obliteration of keratocyte nuclei and corneal stroma. Mild, moderate, or severe was given a score of 1, 2, or 3, respectively. A clear, normal mouse cornea in which no or few inflammatory cells were seen was given a score of 0.

### Reverse transcription-polymerase chain reaction of major inflammatory cytokines

The corneas were excised within the 3 mm central area and stored at −80 °C until assayed. Total RNA was extracted from each excised cornea using the NucleoSpin RNA II System (Macherey-nagel, Düren, Germany) according to the manufacturer’s protocol. Total RNA was quantified by ultraviolet spectrophotometer measurement of OD260 and OD280. To eliminate contamination with genomic DNA, the RNA samples (1 μg) were treated with DNase I and subjected to reverse transcription at 42 °C for 60 min in 40 μl of reaction mixture using the first-strand cDNA synthesis kit (BBI, Toronto, Canada). The primer pairs and product lengths are listed in Table 1. The PCR conditions for an initial step were denaturing at 94 °C for 1.5 min and for the remaining 35 cycles were denaturing at 94 °C for 30 s, annealing at 55 °C for 30 s, and extending 72 °C for 1 min. Reverse transcription polymerase chain reaction (RT–PCR) products were separated by 2% agarose gel electrophoresis, detected by ethidium bromide staining, and analyzed using Image J software. The relative mRNA level was obtained by comparing the optical density of cytokine-specific bands to the internal standard (*GAPDH*).

**Table 1:** PCR primers for cytokines and chemokines.

**Factors**	**Primer sequences (F)**	**Primer sequences(R)**	**Size of product (bp)**
*MIP-2*	AGTGAACTGCGCTGTCAATG	TTCAGGGTCAAGGCAAACTT	153
*KC*	GCTGGGATTCACCTCAAGAA	TGGGGACACCTTTTAGCATC	169
*IL-1β*	GCCCATCCTCTGTGACTCAT	AGGCCACAGGTATTTTGTCG	230
*IL-6*	AGTTGCCTTCTTGGGACTGA	TCCACGATTTCCCAGAGAAC	159
*GAPDH*	GGTGAAGGTCGGTGTGAACGGA	TGTTAGTGGGGTCTCGCTCCTG	439

Primers for cytokines and chemokines used for reverse transcriptional PCR.

### ELISA of major inflammatory cytokines

Protein levels of the four major inflammatory cytokines were detected using ELISA kits (Jingmei Biotech Co Ltd, Shanghai, China). The corneas were excised within the 3 mm central area and stored at −80 °C until assayed. The samples were weighed, thawed, minced, homogenized with a glass pestle in 1.5 ml of PBS, sonicated for 30 s, and clarified by centrifugation at 16,000x g for 15 min at 4 °C. Samples were diluted in a ratio of 1:5 in a sample diluting buffer and were added to the wells in duplicate. Samples were analyzed according to the manufacturer’s instructions. The results were analyzed as picograms of cytokine per milligram of corneal tissue (pg/mg).

### Statistical analyses

SPSS 11.5 software for Windows (SPSS Inc., Chicago, IL) was used for statistical analysis. The results from RT–PCR, ELISA, and clinical scoring were analyzed using one-way ANOVA to test for statistically significant differences. The expression levels of KC and MIP-2 proteins before and after the antibody intervention experiment were analyzed using paired-sample *t*-test. A p value of less than 0.05 was considered significant. All experiments were repeated at least twice to ensure reproducibility.

## Results

### Course of fungal keratitis

**Figure 1:**
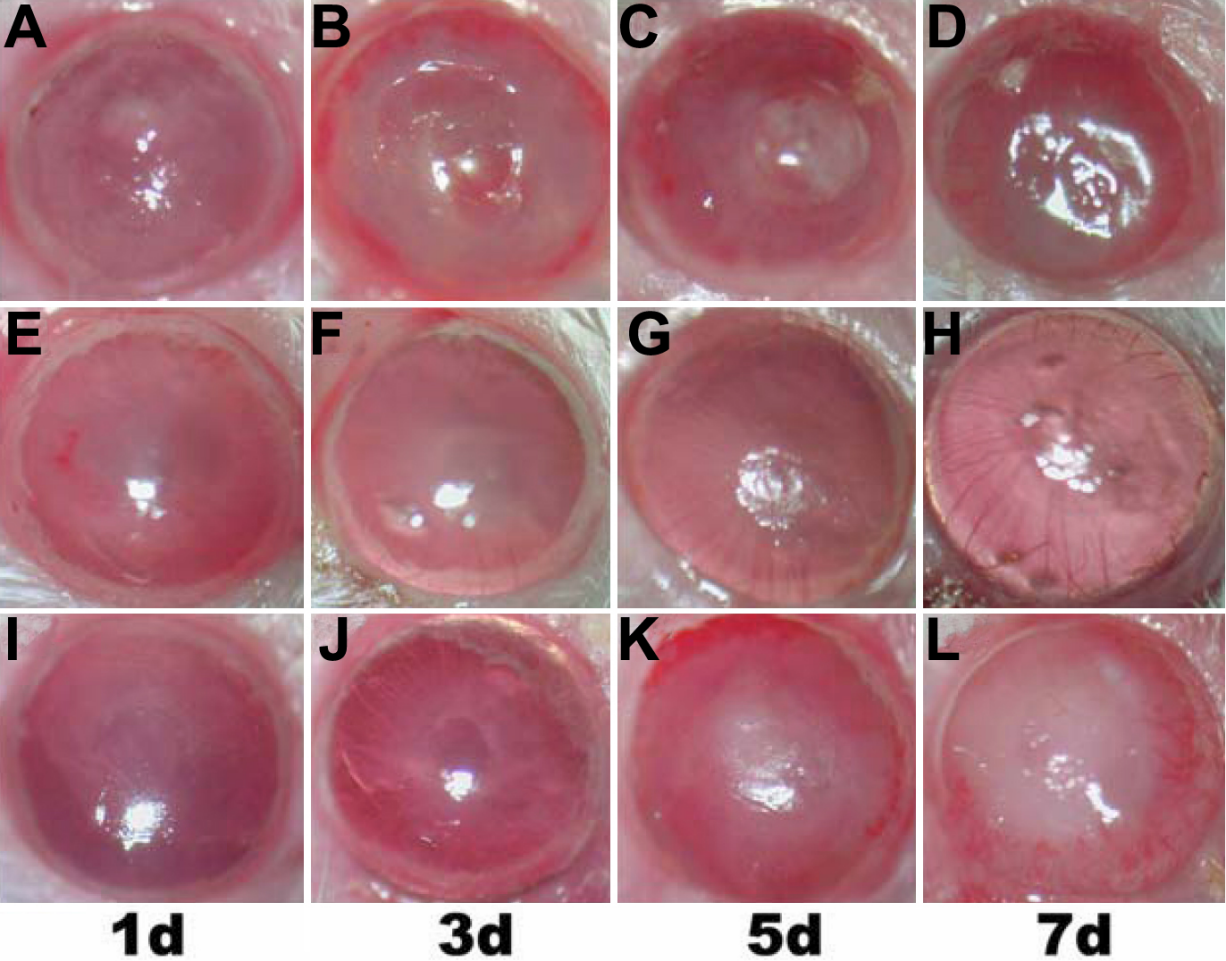
Clinical progression of fungal keratitis induced by *A. fumigatus* in mice. The images in the first row (**A**, **B**, **C**, and **D**) show disease process one, three, five, and seven days, respectively, post-infection in the infected group. The images in the second row** (E**, **F**, **G**, and **H**) show the disease process one, three, five, and seven days, respectively, post-infection in the MIP-2 treated group. Here, the corneal lesion was reduced at all time points. The images in the third row (**I**, **J**, **K**, and **L**) show the disease process at one, three, five, and seven days, respectively, post-infection in the IL-1β treated group. The corneal lesion was significantly reduced on day 1 and 3 post-infection.

**Figure 2:**
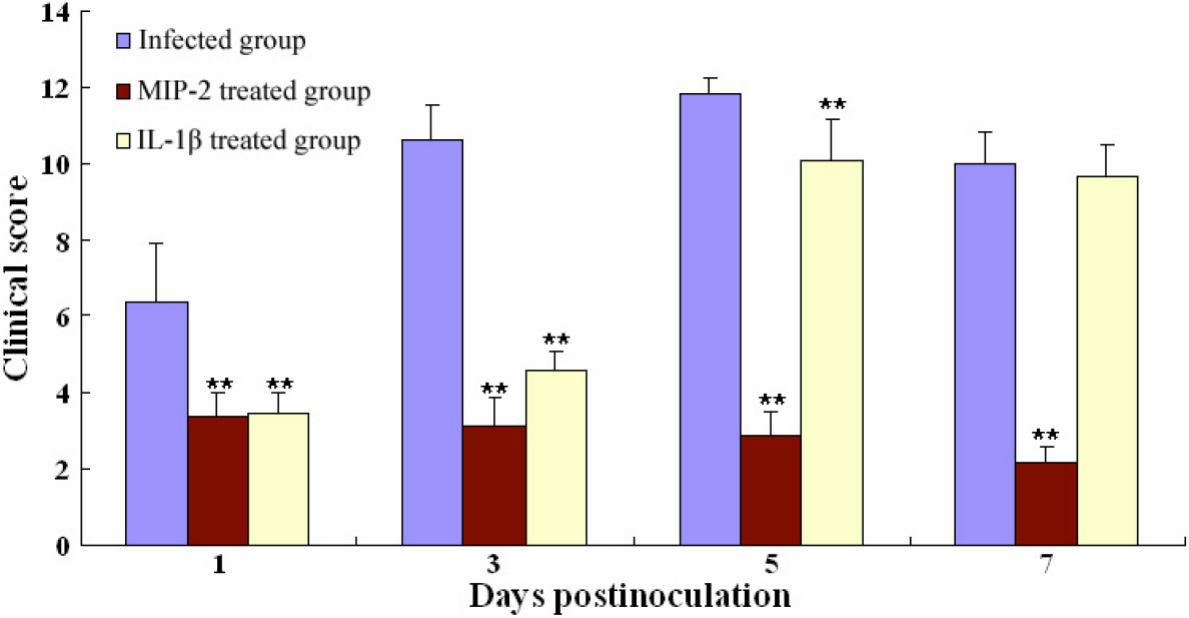
Clinical scores of *A. fumigatus*-induced keratomycosis in mice. The clinical scores significantly decreased at all time points in the MIP-2 treated group and on days 1, 3, and 5 in the IL-1β treated group. The asterisks indicate significance levels assessed via ANOVA followed by post-hoc tests: one asterisk indicates p<0.05 and two asterisks indicate p<0.01.

All of the eyes inoculated (120/120) developed a focus of infection in their corneas the day after the operation and eventually developed complete lesions. The clinical features of the mice infected with *A. fumigatus* and *C. albicans* were similar. The duration of infection with *A. fumigatus* and *C. albicans* was about 10 days. The features manifested most evidently one day after inoculation and varied significantly between the treated group and infected group. In the infected group, the corneas were characterized by severe inflammation, marked edema, ulceration, and neovascularization. In the MIP-2 treated group, the infected lesions were significantly reduced postoperatively, and the lesions were without corneal ulceration or necrosis and instead displayed only corneal epithelial defects, shallow-matrix edema, and minimal collagen degeneration. The cornea remained transparent, and the anterior chamber and iris texture were clearly visible one and three days after infection. Seven days after surgery, the corneal epithelium had healed, corneal transparency was recovered, and no limbal neovascularization was apparent. In the IL-1β treated group, the infected lesions were significantly reduced one and three days after surgery and increased gradually later. The course of FK and clinical scores of each eye at the four time points are shown in Figure 1 and Figure 2 (some data not shown).

### Histology

**Figure 3:**
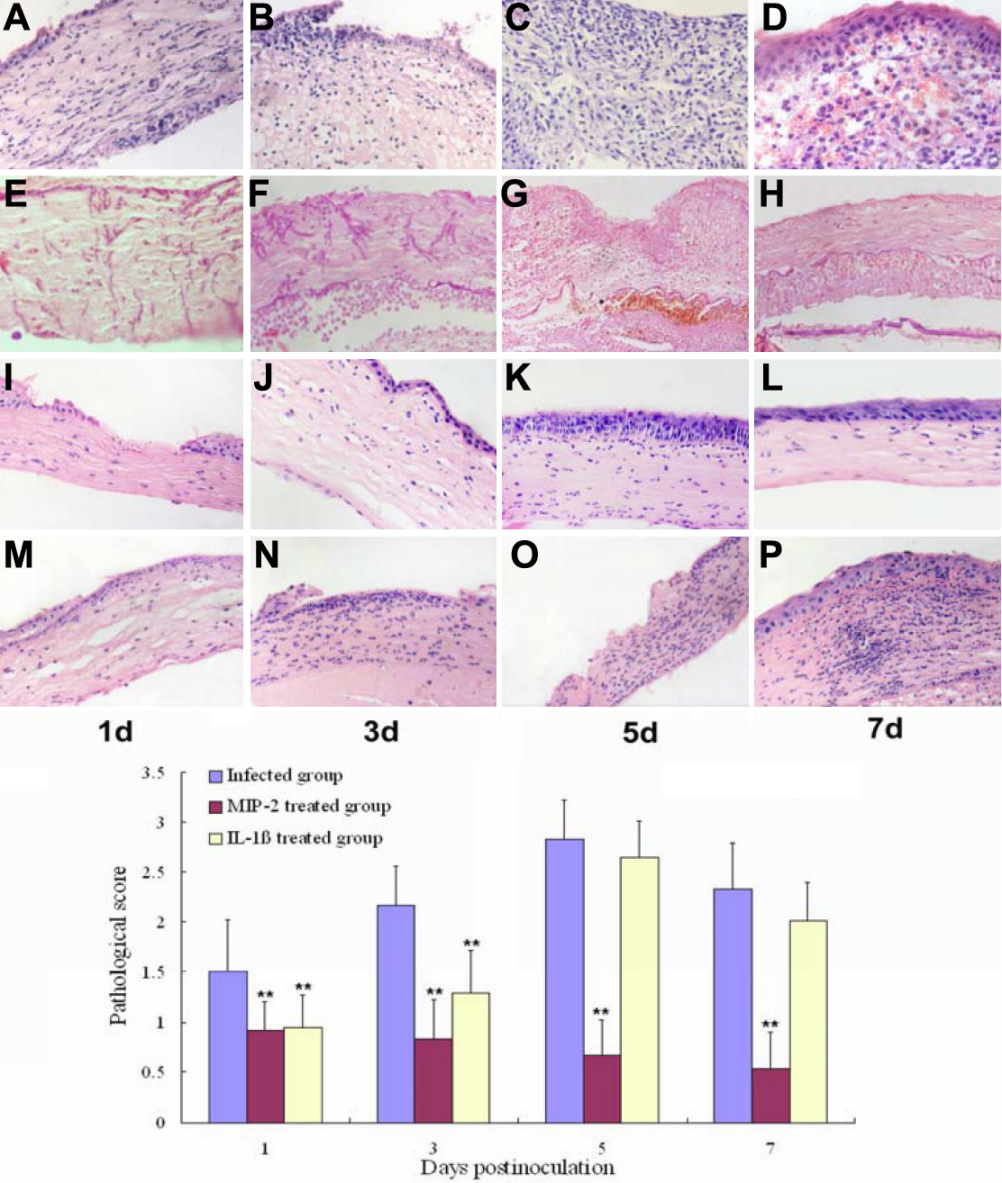
Histopathology of *A. fumigatus*-induced keratomycosis in mice. **A**, **B**, **C**, and **D** show the inflammatory changes on days 1, 3, 5, and 7 post-infection in the infected group (magnification 400X). **E**, **F**, **G**, and **H** show the fungal hyphae in the infected group (magnification 400X). **I**, **J**, **K**, and **L** represent the inflammatory changes in the MIP-2 treated group. Here, the inflammatory responses were significantly reduced at all time points (magnification 400X). **M**, **N**, **O**, and **P** represent the inflammatory changes in the IL-1β treated group. The inflammatory responses were significantly reduced on days 1 and 3 post-infection (magnification 400X). The bar chart shows the pathological scores obtained in a semi-quantitative way. The asterisks indicate significance levels assessed via ANOVA followed by post-hoc tests: one asterisk indicates p<0.05 and two asterisks indicate p<0.01.

The histopathologic features and pathological scores were rather different between the treated group and infected group (Figure 3, some data not shown).

The inflammatory responses observed in the infected group demonstrated minimal inflammatory cell infiltration in the early stage of infection, which increased gradually with disease progression and was accompanied by severe corneal tissue damage, epithelial defects, and corneal ulceration. The inflammatory responses were most severe five days after surgery, and inflammatory cell numbers significantly decreased as the corneal epithelium gradually recovered. Corneal neovascularization occurred in seven days after surgery.

Following treatment with MIP-2 polyclonal antibody, the inflammatory response and pathological scores were significantly reduced (p<0.01) in comparison to the inflammation observed in the infected group. Observations included a lack of central corneal epithelia, clear stromal background and corneal cell nuclei, sparse PMNs scattered in the shallow corneal matrix, and an absence of inflammatory cell leakage into the anterior chamber. After treatment with IL-1β polyclonal antibody, the inflammatory responses and pathological scores were significantly reduced one and three days post-infection (p<0.01). After five days of infection, the inflammatory responses and pathological scores were similar to those observed in the infected group. There was no difference in the number of fungal hyphae between the MIP-2 treated group and IL-1β treated group in comparison to the infected group by PAS staining. The presence of hyphae growth was observed one and three days post-infection, and no hypha in the corneal matrix was observed five and seven days post-infection.

### mRNA expression of major inflammatory cytokines

**Figure 4:**
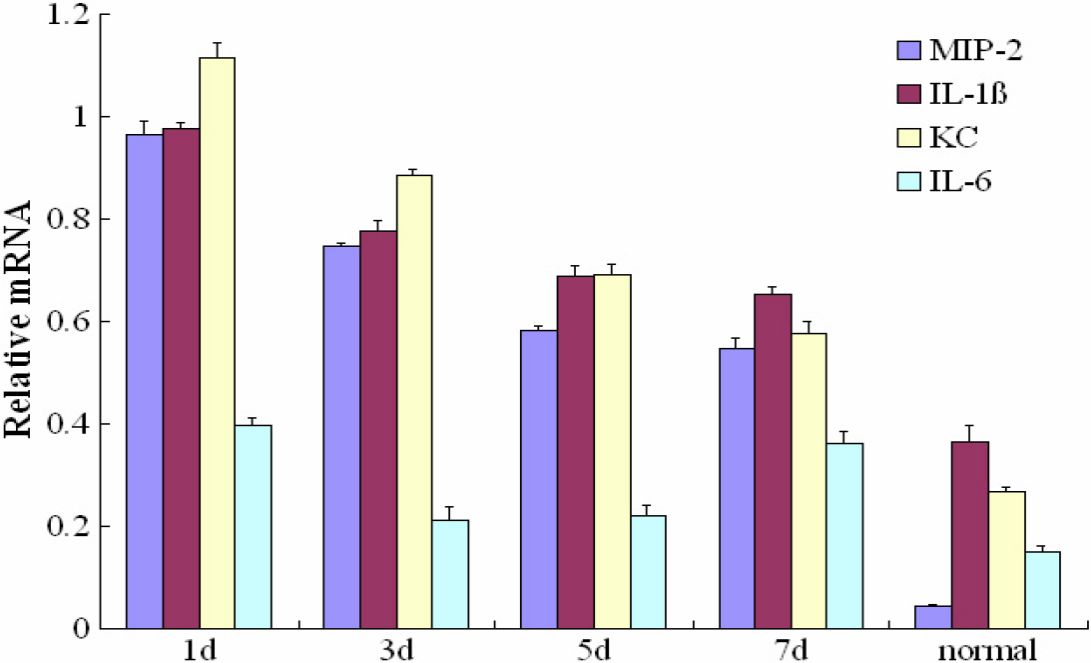
Detection of *MIP-2*, *KC*, *IL-1β*, and *IL-6* mRNA in *A. fumigatus*-induced mouse cornea. The bar chart shows the relative mRNA levels of *MIP-2*, *IL-1β*, *KC*, and *IL-6*. The relative mRNA levels were obtained by comparing the optical densities of cytokine-specific bands to the density of an internal standard, *GAPDH*. *MIP-2*, *KC*, *IL-1β*, and *IL-6* were all found to be significantly upregulated in the infected group compared to the normal control group (p<0.01)

*MIP-2*, *KC*, *IL-1β*, and *IL-6* were all found to be upregulated significantly at the mRNA level in the infected group compared to the normal control group (p<0.01; Figure 4). The mRNA expression levels of *IL-1β* and *KC* were higher than those of *IL-6* and *MIP-2*. The peak expression levels of *MIP-2*, *KC*, and *IL-1β* occurred at one day post-infection, and the mRNA expression levels gradually decreased later. *IL-6* expression levels were lowest among the four cytokines, and the peak expression levels occurred one and seven days post-infection.

### Protein expression of major inflammatory cytokines

**Figure 5:**
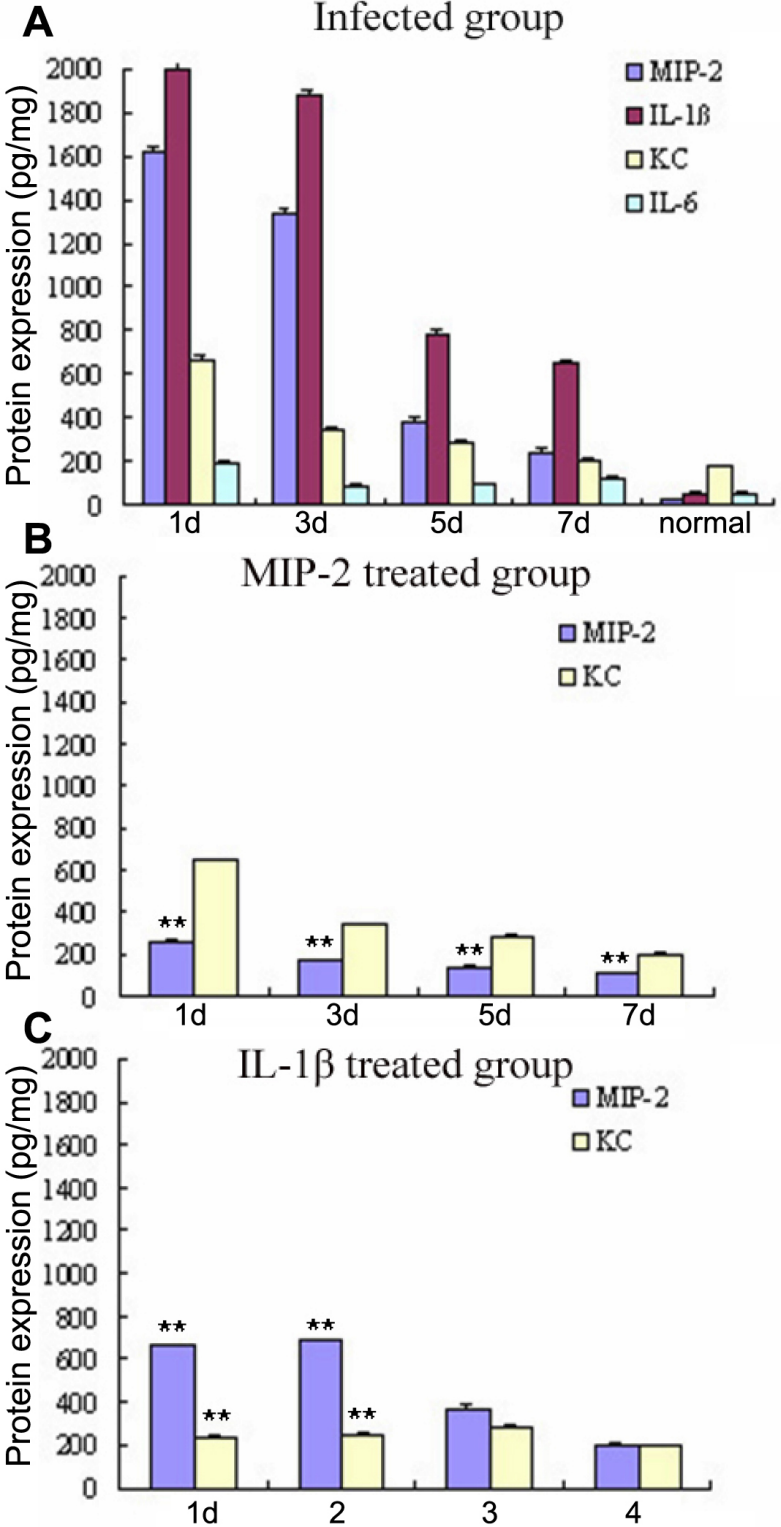
ELISA analysis of MIP-2, KC, IL-1β, and IL-6 protein expression in *A. fumigatus*-induced mouse cornea. **A** shows protein expression levels of MIP-2, KC, IL-1β, and IL-6 for the infected group on days 1, 3, 5, and 7 post-infection. **B **shows protein expression levels of MIP-2 and KC in the MIP-2 treated group. Here, the MIP-2 protein expression levels were lower compared to the infected group. **C **shows protein expression levels of MIP-2 and KC in the IL-1β treated group. This bar chart illustrates that the protein expression levels of MIP-2 and KC on days 1 and 3 were lower compared to the infected group on those same days. The asterisks indicate significance levels assessed via ANOVA followed by post-hoc tests: one asterisk indicates p<0.05 and two asterisks denote p<0.01.

The mRNA data were confirmed by ELISA analysis (Figure 5A), which also showed that the protein levels of MIP-2, KC, IL-1β, and IL-6 were upregulated significantly in the corneas of the infected group than those of the normal control group (p<0.01). In the infected group, the IL-1β protein expression levels of the four cytokines were were highest followed by MIP-2, KC, and IL-6, respectively, which varied slightly from the semi-quantitative analysis of mRNA expression. The trend of protein expression of the four cytokines with the time change was the same as the cytokine mRNA expression and the protein expression levels peaked at one day after surgery.

Protein expression levels of MIP-2 and KC of the MIP-2 and IL-1β treated groups are shown in Figure 5B,C. The results showed that MIP-2 protein expression levels were reduced significantly (p=0.000) one, three, five, and seven days post-infection in the MIP-2 treated group compared to the infected group and that the KC protein expression levels decreased slightly (p>0.05). The protein expression levels of MIP-2 and KC decreased significantly (p=0.000) one and three days post-infection in the IL-1β treated group compared to the infected group, but five and seven days post-infection, the expression of MIP-2 and KC was not significantly different (p>0.05).

## Discussion

This study is the first to demonstrate the expression and potential role of the four major inflammatory cytokines (MIP-2, KC, IL-1β, and IL-6) in the pathogenesis of FK at the gene and protein level. Moreover, among the inflammatory cytokines detected, expression levels of MIP-2, KC, and IL-1β were closely correlated with disease severity and progression of FK, demonstrating that these factors are the major inflammatory cytokines responsible for the pathogenesis of FK. The inflammatory responses in *A. fumigatus* and *C. albicans* keratitis progressed rapidly at an early stage with a peak time to disease being three to five days and maintenance of disease as far as seven days post-infection. Therefore, expression of the four cytokines in FK reached its peak one day post-infection and then slowly declined. As a result, the infiltration of inflammatory cells rapidly increased during the early stage of infection and perhaps played dual roles of fighting pathogens as well as destroying infected tissue. The above analysis demonstrated that a high expression of the major inflammatory cytokines induces a large number of inflammatory cell infiltrates, which leads to a significant aggravation of corneal lesions.

In an experimental *Pseudomonas aeruginosa* keratitis study, the susceptibility to infection by *Pseudomonas aeruginosa* was shown to involve dysregulation of the local host response including persistence of PMNs and increased levels of IL-1 and MIP-2. Mice resistant to *Pseudomonas* infection rapidly downregulate these responses [21]. The current study shows that when FK progresses, the number of fungal hyphae in the diseased cornea gradually reduced and fungal hyphae were rarely seen five days post-infection, but the inflammatory responses of disease reached a peak at this time. This phenomenon revealed that there may be a possible dysregulation of the local host response in the pathogenesis of FK. At the early stage of the disease (one to three days), the inflammatory responses induced by fungal pathogens is sufficient to resist and kill pathogens and to control the development of disease as long as expression of MIP-2, KC, and IL-1β is maintained or even further increased. During this time, PMN infiltration will continuously increase thus leading to progression of disease and severe tissue necrosis. Evidently, appropriate expression of the major inflammatory cytokines, specifically MIP-2 and IL-1β, play an important role in resisting pathogens and repairing tissues while keeping destructive effects to a minimum, which may be the key in controlling the development of disease and may provide insight into a breakthrough for finding new treatments against FK.

MIP-2 (human homolog IL-8) is an important chemokine in response to bacterial infection, and maintenance of high MIP-2 expression levels is directly related to an increase in corneal PMNs and severity of corneal inflammation. In contrast, timely downregulation of corneal MIP-2 was shown to correlate with a decrease in PMNs in resistant mice and was followed by corneal healing and reestablishment of ocular integrity [16,22]. Activated PMNs produce MIP-2 (mouse) or IL-8 (human), which produces an auto-amplification loop of PMN recruitment and activation at the inflamed sites, and this positive feedback is considered to be one of the potential mechanisms by which MIP-2 could amplify the local inflammatory response of the PMNs [23,24]. KC is also a chemokine and signals to neutrophils, but its role is considered to be much weaker than that of MIP-2, although the concentration of KC is much higher than that of MIP-2 [14]. This study suggests that MIP-2 is the most important chemokine in FK pathogenesis, and maintenance of high levels of MIP-2 protein is directly related to the severity of corneal lesions. Despite high expression of *KC* at the mRNA level, KC is expressed far less than MIP-2 at the protein level. In addition, the expression levels of KC are higher at an early stage but decline rapidly three days post-infection, which is consistent with the phenomenon observed by Kernacki et al. [16] in *Pseudomonas aeruginosa* keratitis in mice. This finding also illustrates that KC is not the key chemokine responsible for the development of FK. Furthermore, when mice were given a subconjunctival injection of MIP-2 polyclonal antibody, the severity of corneal disease was significantly reduced. MIP-2 expression in *A. fumigatus* and *C. albicans* keratitis peaked at the earliest time. Therefore, early intervention by administration of MIP-2 antibody had the greatest impact on the development of FK. The reason for this phenomenon may due to inhibition of the auto-amplification loop.

IL-1β is a cytokine with a wide range of roles and is produced by inflammatory cells as well as by resident corneal cells [17]. Runder et al. [25] discovered that *IL-1β* mRNA and protein expression levels were significantly elevated in mice with *Pseudomonas aeruginosa* keratitis and that expression levels were correlated with the severity of disease. When mice received injections with anti-IL-1β polyclonal antibody, the severity of corneal disease was significantly reduced, and this was accompanied by a reduction in corneal PMN cells, bacterial load, and *MIP-2* mRNA and protein expression. In this study, IL-1β was the proinflammatory cytokine with the highest expression levels in *A. fumigatus* and *C. albicans* keratitis, and the IL-1β expression levels were related closely to corneal disease severity and inflammatory cell infiltration. When experimental mice were subconjunctivally injected with IL-1β polyclonal antibodies, the corneal inflammatory responses were significantly reduced at an early stage (one to three days post-infection). This indicates that IL-1β may play a central role in the pathogenesis of FK and that treatment targeted at the expression of IL-1β may be a potential therapeutic strategy for FK. Although the disease process rebounded at the middle and late stages after treatment, this may be explained by the efficacy of the antibody, which may have disappeared or been deactivated by the later stage, allowing the pathogens to survive and induce the host inflammatory responses. In addition, one and three days after administration of IL-1β polyclonal antibody, the protein expression of MIP-2 and KC significantly decreased in the IL-1β treated group. Five to seven days post-infection, the protein levels of the two cytokines rose markedly but remained lower than the levels observed in the infected group. These data were consistent with rebounding of the disease process at the middle and late stages of infection and also showed that IL-1β played a role in regulating the expression of MIP-2 and KC.

IL-6 is a multifunctional cytokine known to upregulate the immune response thus generating humoral or cellular immune reactions and stimulating liver cells to synthesize acute phase proteins to form an acute phase inflammatory response [26]. IL-6 is critical in the host defense of the cornea during infection with *Pseudomonas aeruginosa*. Effective recruitment of neutrophils into the cornea is dependent on the production of IL-6, and IL-6 has been demonstrated to be a protective factor in corneal infection [27]. In this study, IL-6 was expressed in the corneas of both the infected group and normal mice, but the protein levels were the lowest out of the four inflammatory cytokines measured, which suggests that IL-6 is not the key inflammatory mediator in FK. Based on the confirmed roles of IL-6, a high-level expression of IL-6 at the early stage (one day post-infection) may be induced by the body’s stress response secondary to acute infection. Increased expression of IL-6 at the late stage (seven days post-infection) may play a role in stimulating lymphocyte activation, proliferation, and differentiation, and it may be important in promoting the humoral and cellular immune responses. This hypothesis is supported by the fact that lymphocytes in the body recognize specific pathogenic antigens and generate immune responses through a series of mechanisms that are most active five to seven days after infection. Since the acute phase response and the specific immune response are the body's most important defense mechanisms, IL-6 may also be a protective factor in the pathogenesis of FK.

In brief, this study is an initial exploration into the expression, regulation, and effects of the four major inflammatory cytokines in the inflammation process observed with FK. Based on the analyses presented in this study, MIP-2 is a crucial chemokine for the onset of FK in mice, and IL-1β is the major cytokine responsible for regulation of MIP-2 and KC. Persistent, high-level expression of MIP-2 and IL-1β is an important factor in promoting corneal impairment. In contrast, the expression of IL-6 may play an important role in protecting the mouse against FK. After isolating a crucial player responsible for the course of FK, specific polyclonal neutralizing antibodies may be potentially used to inhibit the major chemokines and cytokines thus effectively relieving the corneal damage. This result is encouraging, and it will be important in the future to explore this information to help develop new therapeutics aimed at the major inflammatory cytokines in FK.
